# Titania Nanofiber Scaffolds with Enhanced Biointegration Activity—Preliminary In Vitro Studies

**DOI:** 10.3390/ijms20225642

**Published:** 2019-11-11

**Authors:** Michalina Ehlert, Katarzyna Roszek, Tomasz Jędrzejewski, Michał Bartmański, Aleksandra Radtke

**Affiliations:** 1 Faculty of Chemistry, Nicolaus Copernicus University in Toruń, Gagarina 7, 87-100 Toruń, Poland; m.ehlert@doktorant.umk.pl; 2 Nano-Implant Ltd., Gagarina 5/102, 87-100 Toruń, Poland; 3 Faculty of Biological and Veterinary Sciences, Nicolaus Copernicus University in Toruń, Lwowska 1, 87-100 Toruń, Poland; kroszek@umk.pl (K.R.); tomaszj@umk.pl (T.J.); 4 Faculty of Mechanical Engineering, Gdańsk University of Technology, Gabriela Narutowicza 11/12, 80-233 Gdańsk, Poland; michal.bartmanski@pg.edu.pl

**Keywords:** titanium alloy, nanofibers, adipose-derived mesenchymal stem cells, wettability, mechanical properties, biological activity

## Abstract

The increasing need for novel bone replacement materials has been driving numerous studies on modifying their surface to stimulate osteogenic cells expansion and to accelerate bone tissue regeneration. The goal of the presented study was to optimize the production of titania-based bioactive materials with high porosity and defined nanostructure, which supports the cell viability and growth. We have chosen to our experiments TiO_2_ nanofibers, produced by chemical oxidation of Ti6Al4V alloy. Fibrous nanocoatings were characterized structurally (X-ray diffraction (XRD)) and morphologically (scanning electron microscopy (SEM)). The wettability of the coatings and their mechanical properties were also evaluated. We have investigated the direct influence of the modified titanium alloy surfaces on the survival and proliferation of mesenchymal stem cells derived from adipose tissue (ADSCs). In parallel, proliferation of bone tissue cells—human osteoblasts MG-63 and connective tissue cells - mouse fibroblasts L929, as well as cell viability in co-cultures (osteoblasts/ADSCs and fibroblasts/ADSCs has been studied. The results of our experiments proved that among all tested nanofibrous coatings, the amorphous titania-based ones were the most optimal scaffolds for the integration and proliferation of ADSCs, fibroblasts, and osteoblasts. Thus, we postulated these scaffolds to have the osteopromotional potential. However, from the co-culture experiments it can be concluded that ADSCs have the ability to functionalize the initially unfavorable surface, and make it suitable for more specialized and demanding cells.

## 1. Introduction

The wide use of long-term implants in various fields of medicine is associated with an increasing demand for bone replacement materials to reconstruct the function of bone tissues and their rapid and effective healing [1,2,3,4,5]. Successful meeting of these requirements depends mainly on the degree of integration between the biomaterial (implant) and the native bone tissue. Hard materials (metals and their alloys) used in biomedical applications must exhibit specific properties in order to promote bone regeneration, proliferation, and osteogenic differentiation, [1,2,3,6,7]. The material suitable for implants should be biocompatible, should possess excellent corrosion resistance, optimal Young’s modulus (similar to bone’s modulus), low weight, high fatigue resistance, and adequate porosity. In addition, it is recommended for such material to be inexpensive and easy to produce. The unique physical and chemical properties of titanium and its alloys make them most frequently used among metallic biomaterials. Titanium is a biocompatible material, but it is not bioactive [4,6,8,9,10]. One of the ways to improve the osseointegration of titanium/titanium alloys implants is the modification of their surface by the fabrication of oxide coatings with a specific structure, architecture, and physicochemical properties. Based on results of earlier investigations, it can be stated that cells are highly susceptible to any changes in morphology, structure, roughness, and surface energy. These factors can directly affect the rate and degree of cell adhesion, proliferation, differentiation, as well as having an impact on cell morphology [8,9,10]. Simultaneously, it should be noted that bone tissue contains a lot of components present at the nanoscale, therefore it is beneficial to create an implant surface, which can imitate this nanoarchitectural hierarchy [11,12,13]. Considering the geometrical properties of collagen and hydroxyapatite crystals, bone cells should react better and more intensively on substrates characterized by surface topography and architecture at the nanometric level [13,14,15]. The surface modification of the titanium/titanium alloy implant was intended to facilitate the production of coatings with a strictly defined nanotopography, e.g., nanofibers, nanorods, nanotubes, nanoteeths, nanowires, which promotes the process of its binding with the bone tissue [16,17,18,19,20,21]. One of the effective methods leading to the formation of a bioactive surface material is the use of the chemical oxidation process [22,23]. Chemical treatments of titanium and its alloys are mainly based on chemical reactions occurring on the interfacial surface between titanium and the solution. The most common chemical procedures include acid, alkaline, H_2_O_2_, heat, and passivation treatments [24,25]. They are carried out in order to remove native oxides and impurities from the surface. This method may also lead to obtaining layers with increased biocompatibility, bioactivity, and bone conductivity [23,25]. In our research, among many available ways of preparing bioactive titanium surfaces, we have focused on applying of various acids and further oxidation in the presence of H_2_O_2_. Acid etching usually leads to a thin oxide layer on the surface, mainly TiO_2_ (<10 nm) [25]. The application of hydrogen peroxide as an oxidant does not cause any external contamination in the reaction system. Under the influence of H_2_O_2_, the production of titanium peroxide gel is expected on the sample surface [18,25,26]. As a result of immersion of titanium foil in H_2_O_2_ solution, metallic titanium reacts with H_2_O_2_ molecules to form Ti-OH groups on the surface of titanium [26,27,28]. It can be expected that the longer the process is carried out, the rougher the layer will become [26]. 

The effectiveness of osteointegration and healing processes are caused by demands from the nanostructured surface of the biomaterial to stimulate, inter alia, osteogenic differentiation of mesenchymal stem cells (MSCs) [10,12,29,30,31]. Stem cells become activated when an injury occurs and are recruited to the injury site to support tissue repair. When a biomaterial is implanted, the body reacts similarly and stem cells are recruited to the implant site. Therefore, stem cell interactions with biomaterials are critical for the long-term success of medical devices. The most widely used source of stem cells in scientific research and clinical practice is bone marrow [32]. However, the procedure of bone marrow collection is painful, has certain complications, and provides a limited amount of multipotent cells [33,34,35]. An alternative, attractive, and easily accessible source of progenitors is the stromal vascular fraction (SVF) of adipose tissue. The SVF is a heterogeneous mixture containing many types of cells, including blood cells, fibroblasts, endothelial cells, adipocytes, as well as adipose-derived mesenchymal stem cells (ADSCs) [31,36,37,38]. Stem cells obtained from subcutaneous adipose tissue have a multipotent differentiation capacity comparable to that of bone marrow stem cells [1,11,31,39,40,41]. In addition, mesenchymal stem cells of adipose tissue expand easily in the cell culture and age slowly. Therefore, they are presently considered as one of the most promising sources of cells to be applied in tissue engineering to repair, replace or regenerate tissues and organs damaged by diseases or injuries. To date, very few studies have investigated the adhesion, proliferation, and differentiation of ADSCs on titania surfaces. 

Another important issue to prevent unwanted clinical complications during implantation is maintaining a permanent connection between the connective tissue and the implant [1,11,30,40,41,42,43,44]. For this reason, we used the co-culture system of ADSCs with osteoblasts and fibroblasts in the present study to verify the efficacy of the engineered bone tissue integration. Rozila and colleagues, among others, used a similar research approach testing osteogenic potential of human adipose-derived stem cells co-cultured with human osteoblasts on polymeric microfiber scaffolds [45]. Mesenchymal stem cells, similarly to fibroblasts, are also suggested to support wound healing because they produce multiple growth factors and cytokines, which are of major interest in wound healing processes [46]. Therefore, in the present paper we have examined if ADSCs might stimulate fibroblast proliferation, especially since several studies have demonstrated the paracrine effects of ADSCs on fibroblasts. For example, Park et al. showed in an animal model that ADSCs and their conditioned media could stimulate collagen synthesis and promote the migration of fibroblasts to wounds [47]. Shen et al. demonstrated that ADSCs promote proliferation in young and aged fibroblasts through a paracrine mechanism [48].

The main objective of presented study was to investigate in vitro the ability of titanium dioxide nanofibers, produced in the process of Ti6Al4V alloy surface chemical oxidation, to support the growth, adhesion, proliferation, and differentiation of mesenchymal stem cells of adipose origin (ADSCs). We have also determined the growth possibility of cell co-cultures (fibroblasts/ADSCs, and osteoblasts/ADSCs) on nanofibrous coatings. So far, researchers have not concentrated on functionalization of TiO_2_ nanofibrous surfaces with adipose mesenchymal stem cells and on the use of ADSCs as a highly bioactive layer supporting bone tissue cells - osteoblasts MG-63 and connective tissue cells - fibroblasts L929. The results of our studies provide preliminary but valuable information about cell biology and interactions with the surface modified implants, which can be beneficial for everyday surgical practice (maxillofacial, dental, plastic, and orthopedic).

## 2. Results

### 2.1. Morphology and Structure Characterization of Titania Nanocoatings

**Figure 1:**
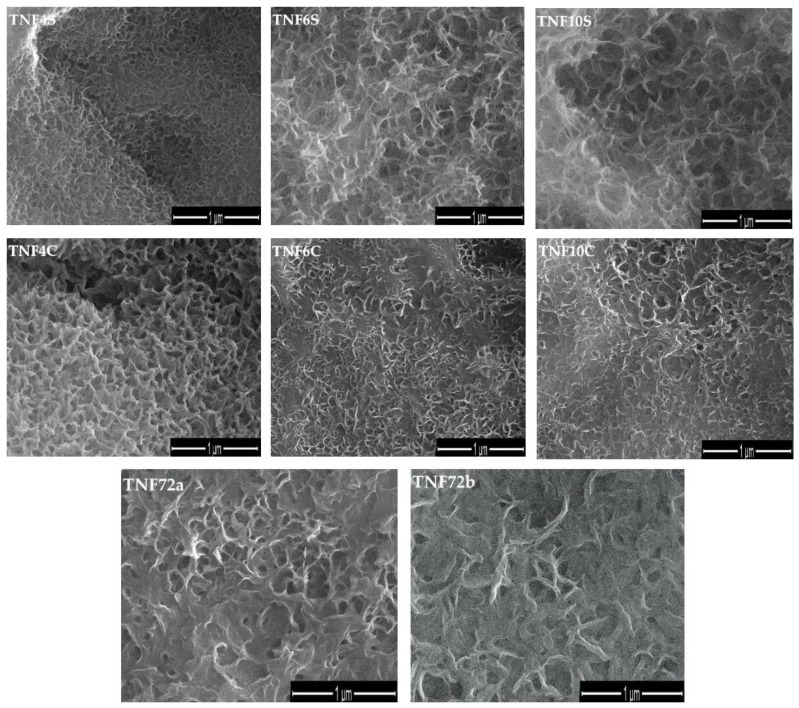
Scanning Electron Microscopy (SEM) images of titania nanofibers (TNF) on Ti6Al4V alloy surface.

Applying the direct oxidation method of Ti6Al4V foil surface led to the formation of titanium dioxide coatings, which consist of nanofibers (TNFs). Analysis of SEM images revealed a close relationship between the applied heating way (in an incubator (TNF4S-10S) or under a reflux condenser (TNF4C-10C), the time of the process, and morphology of formed TNF coatings (Figure 1). Uniform coatings without cracks and gaps were obtained for all samples. It was found that the fibrous nature of the samples heated under reflux was more visible compared to those heated in the incubator. Moreover, the analysis of SEM images showed that an extension of heating time led to obtaining a more interlinked, nanofibrous morphology. Differences between morphology of the TNF4C sample and TNF6C, TNF10C ones are a good example of this (Figure 1). 

In order to distinguish the systems formed on the Ti6Al4V surface heated in an incubator (TNF4S-10S) and reflux cooler (TNF4C-10C) from those formed during its oxidation with 30 wt % H_2_O_2_ solution at 358K for 72 h plate, the latter system was named as nanofibers 72 (TNF72a-b). After the process of chemical oxidation, the deposition of a homogeneous white layer on the surface of samples was observed. This layer was removed by ultrasonic cleaning. The morphology of nanofibers obtained (TNF72a-b) is presented in Figure 1. 

**Figure 2:**
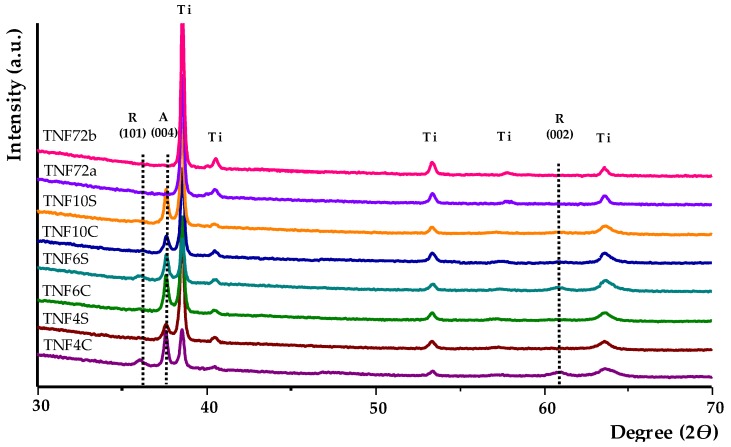
X-ray diffraction spectra of produced Ti6Al4V/TNF samples (lines marked as Ti are assigned to the titanium of titanium alloy substrate).

The structure of produced coatings was determined by X-ray diffractometry (XRD) and registered spectra are presented in Figure 2. Analysis of these data shows that the TNF72 samples are amorphous (no anatase or rutile phase was observed). According to these data the TNF72 samples are amorphous, while TNF4-TNF10 ones are polycrystalline systems. The presence of the signal at 36.0° (101) and 61.3° (002) on the GIXRD spectra of TNF4C-10C and TNF4S-10S, points to the formation of rutile form of TiO_2_ nanofibers. At the same time, the existence of the signal at 37.6° (004) was attributed to the anatase crystalline phase. The diffraction lines, which were found in XRD spectra of TNF4C-10C and TNF4S-10S, are in agreement with the literature data for TiO_2_ rutile and anatase phases [49,50,51,52,53]. 

### 2.2. The Wettability and Surface Free Energy

**Figure 3:**
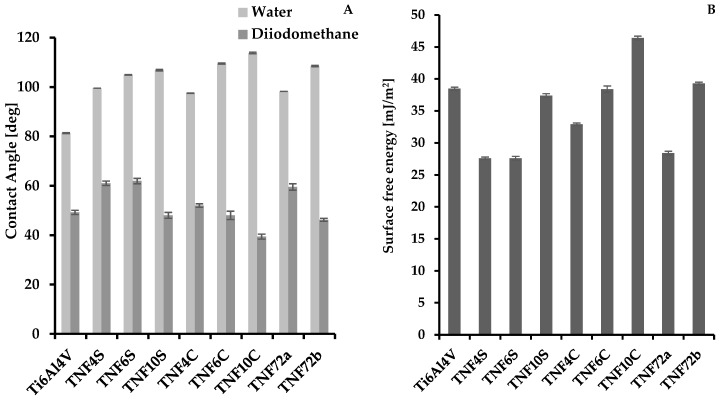
The values of contact angles for water and diiodomethane (**A**), and surface free energy of Ti6Al4V/TNF samples (**B**).

The results of contact angles measurements for water and diiodomethane, and also changes of surface free energy value (SFE) of Ti6Al4V/TNF are presented in Figure 3 and in Table S1. Contact angle differences were attributed to the surface properties, in particular the surface roughness, which affect the contact angle of a water and diiodomethane droplet on each Ti6Al4V surface. According to data presented in Figure 3, it can be stated that samples surfaces indicate a clear hydrophobic character. In the case of Ti6Al4V/TNF4C-10C and Ti6Al4V/TNF4S-10S hydrophobicity increases from TNF4 to TNF10, i.e., parallel with the elongation of the chemical oxidation time of Ti6Al4V surface. When comparing the results obtained for TNF72a and TNF72b, we observed a higher hydrophobicity for the oxidation mixture activated sample (i.e., TNF72b). The free surface energy (SFE) of the produced coatings was calculated using the Owens-Wendt method. This method required the wetting angles be measured for polar liquid—water and dispersion liquid—diiodomethane (Figure 3A and Table S1). The SFE calculations for Ti6Al4V/TNF4-10 samples showed that their values change in the narrow range, i.e., from SFE = 27.6 (mJ/cm^2^) up to SFE = 37.4 (mJ/cm^2^) for Ti6Al4V/4S-10S and from SFE = 32.9 (mJ/cm^2^) up to SFE = 46.4 (mJ/cm^2^) for Ti6Al4V/TNF4C-10C (Figure 3B and Table 1). In the case of Ti6Al4V/TNF72 samples, the SFE value was 28.4 (mJ/cm^2^) for TNF72a and 39.3 (mJ/cm^2^) for TNF72b (Figure 3B and Table S1).

### 2.3. The Roughness of the of Titania Nanocoatings

**Figure 4:**
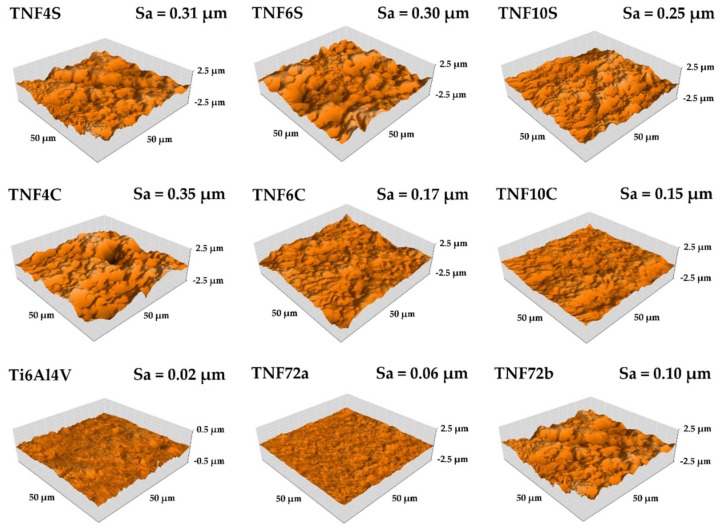
The Atomic Force Microscopy (AFM) topography of Ti6Al4V and TNF samples with Sa parameter values.

The results of AFM topography with a value of area roughness parameter Sa of Ti6Al4V and modified specimens were presented in Figure 4. The lowest value of Sa parameter was obtained for the reference Ti6Al4V specimen. As demonstrated by the results, the oxidation process of the titanium alloy surface increased the roughness for all tested specimens. The same effect were reported in previous research [16,17]. For specimens heated in an incubator (TNF4S-10S) and under a reflux condenser (TNF4C-10C) with the increase of oxidation time (4–10 h), the roughness decrease was noticed. Significant decrease of Sa parameters with increased time of oxidation was obtained for specimens TNF4-10C from 0.35 µm (TNF4C) to 0.17 µm (TNF6C) and 0.15 µm (TNF10C). For samples from groups TNF4-10S no significant decreases in roughness were obtained, but these decreases also occurred. For specimens obtained after oxidation in 2 M HF solution for 10s (TNF72a) and in a 1:4:5 mixture of HF:HNO_3_:H_2_O, the increase of the roughness compared with reference (polished Ti6Al4V sample) from 0.02 µm to 0.06 µm (TNF72a) and 0.10 µm (TNF72a) was observed.

### 2.4. The Nanomechanical Properties of the Titania Nanocoatings

The relevant nanomechanical properties, such as hardness (H) and Young’s Modulus (E), and nanoindentation properties (maximum depth of indentation) are presented in Table 1. To determine resistance to wear resistance and to plastic deformation, the parameters H/E and H^3^/E^2^ were calculated and reported in Table 1. The intimately connected H/E ratio to wear resistances have been shown by Matthews and Leyland [54]. Decreases of the hardness and Young’s Modulus for all tested samples compared with reference Ti6Al4V (10.94 GPa and 212.48 respectively) were observed. Also any increase of hardness and Young’s Modulus a caused decrease of the maximum depth of indentation, hence the lowest value (472 nm) was obtained for Ti6Al4V polished foil. The improvement of mechanical properties resulted in an increase of wear resistance (the H/E ratio) and resistance to plastic deformation H^3^/E^2^. The highest mechanical properties were obtained for Ti6Al4V and TNF72b specimens and therefore the wear resistance values were the highest (0.0513 and 0.0421 for Ti6Al4V and TNF72b, respectively). The significant deviations of the obtained values are characteristic of accurate nanoindentation measurements and they were also noticed previously [55,56]. In case of biomaterials, the most important mechanical properties related to their suitability for cell growth is Young’s Modulus. For TNF4S-10S samples, it was observed that the increase oxidation process time initially decreased the Young’s Modulus by about 30% (from 170.85 to 133.85 GPa) and then their increase by about 2% (from 133.85 to 136.54 GPa). For TNF4C-10C samples initially the effect of elongation of oxidation time for Young’s Modulus was insignificant but for specimens TNF6C and TNF10C E, a value decrease of about 20% was noticed (from 165.11 up to 133.22 GPa). 

The adhesion properties are presented in Table 2. The maximum force at which complete delamination of tested coatings occurred was determined by critical force and corresponding friction force was determined as the critical friction force. For TNF4C-10C specimens the increase of coatings adhesion with increase of time of process was observed. The increase in process time for TNF4-10S samples initially caused a decrease in adhesion by 35% (from 164.20 to 107.40 mN) and next by an insignificant increase to 116.69 mN. The obtained results of nanoscratch-test properties correlated with results obtained in nanoindentation tests (H/E and H^3^/E^2^ ratios), the highest adhesion was (205.15 GPa of critical force) for TNF72b specimens, which was characterized by the highest value of H/E and H^3^/E^2^ ratios.

### 2.5. Cell Proliferation Determined by the MTT Assay

**Figure 5:**
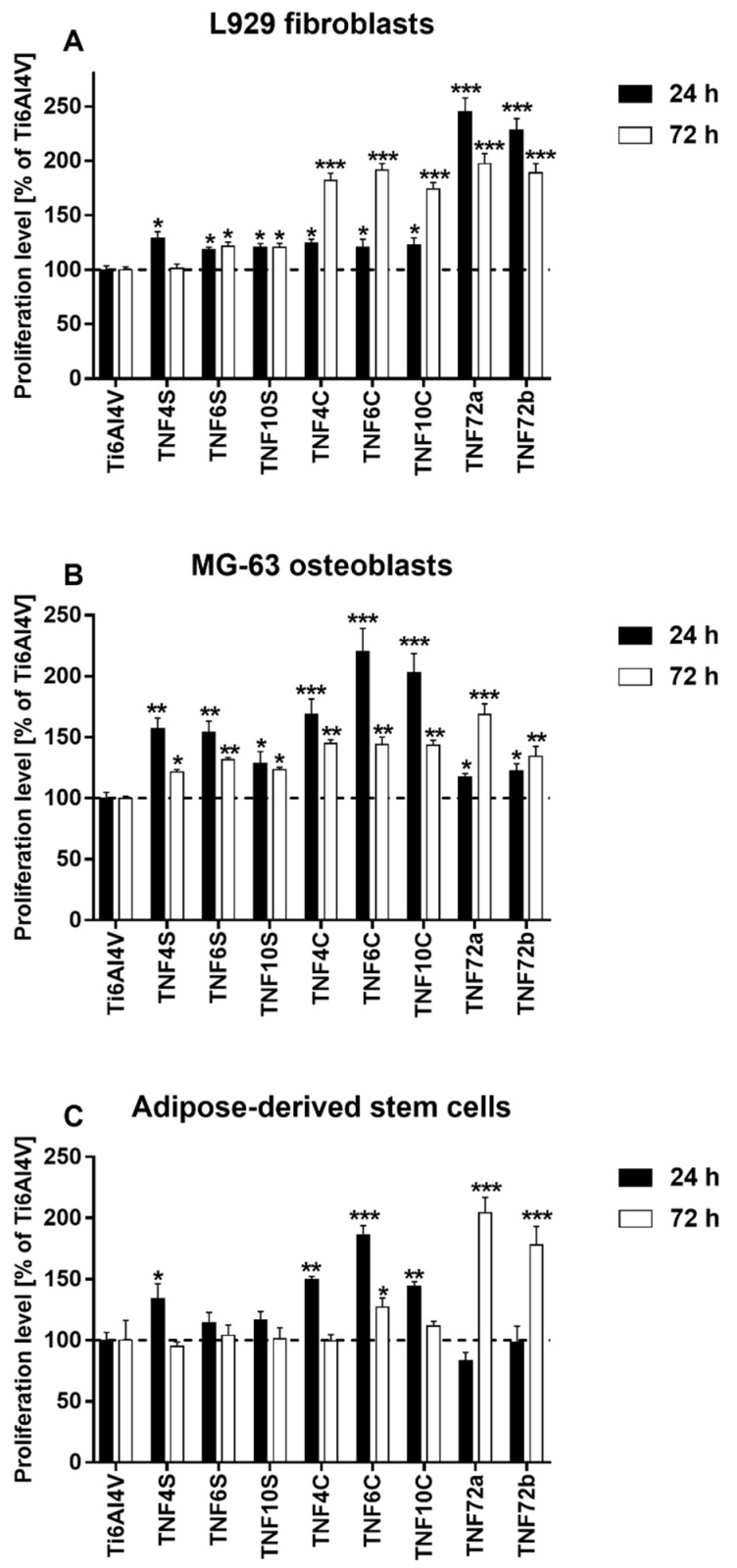
Proliferation level of L929 murine fibroblast cells (**A**), human osteoblast-like MG 63 cells (**B**) and human adipose-derived mesenchymal stem cells (**C**) growing on the surface of fibrous nanocoatings, in comparison with the reference Ti6Al4V alloy foils. Cell viability was assessed using the MTT assay after 24 and 72 h of culture. The results are expressed as percentage of the cells cultured on the reference Ti6Al4V alloy foils (served as 100%). The percentage values are expressed as means ± SEM of four independent experiments. Asterisks indicate significant differences at the appropriate time (after 24 or 72 h) between the level of cell viability on the surface of the tested specimens compared with the reference Ti6Al4V alloy foils (Ti6Al4V) (*** *p* < 0.001, ** *p* < 0.01; * *p* < 0.05). The horizontal lines show the proliferation level of the control cells growing on the reference Ti6Al4V alloy foils.

The proliferation levels of L929 fibroblasts, MG-63 osteoblasts, and adipose-derived mesenchymal stem cells growing on the surface of fibrous nanocoatings were evaluated based on the results of the MTT assay and presented in Figure 5. It is worth noticing that all tested nanolayers provoked a higher proliferation level of L929 fibroblasts (Figure 5A) as well as MG-63 osteoblasts (Figure 5B). This phenomenon was observed after 24 h and 72 h of culture (except for the L929 fibroblasts cultivated on the TNF4S samples for 72 h). Analysis of these data revealed that TNF72a and TNF72b specimens induced the highest L929 cells viability after 24 h (245.2 ± 12.5% and 228.6 ± 10.3%, respectively). In contrast, a longer cultivation time provoked the greatest viability level of L929 cells not only for TNF72a and TNF72b samples (197.6 ± 8.9% and 188.8 ± 8.8%, respectively), but also for TNF4C, TNF6C, and TNF10C nanolayers (182.2 ± 6.5%, 191.1 ± 6.5%, and 174.2 ± 5.8%, respectively). These three specimens also stimulated the highest survival rate of MG-63 osteoblasts (168.6 ± 12.8%, 220.1 ± 19.3%, and 202.9 ± 15.8%, respectively) after 24 h of culture. After 72 h, the highest MG-63 cells viability was observed, especially in case of TNF72a samples (168.7 ± 8.6%). In the case of adipose-derived mesenchymal stem cells, four nanolayers: TNF4S, TNF4C, TNF6C, and TNF10C improved the cell viability during the initial 24 h. Among them, only TNF6C significantly increased the cell viability level after 72 h of culture. On the other hand, TNF72a and TNF72b specimens turned out to be beneficial for ADSCs proliferation. The highest cell viability, 204.6 ± 12.5% of control on the TNF72a nanolayer and 178.1 ± 15.2% on the TNF72b nanolayer, was observed after 72 h (Figure 5C). 

**Figure 6:**
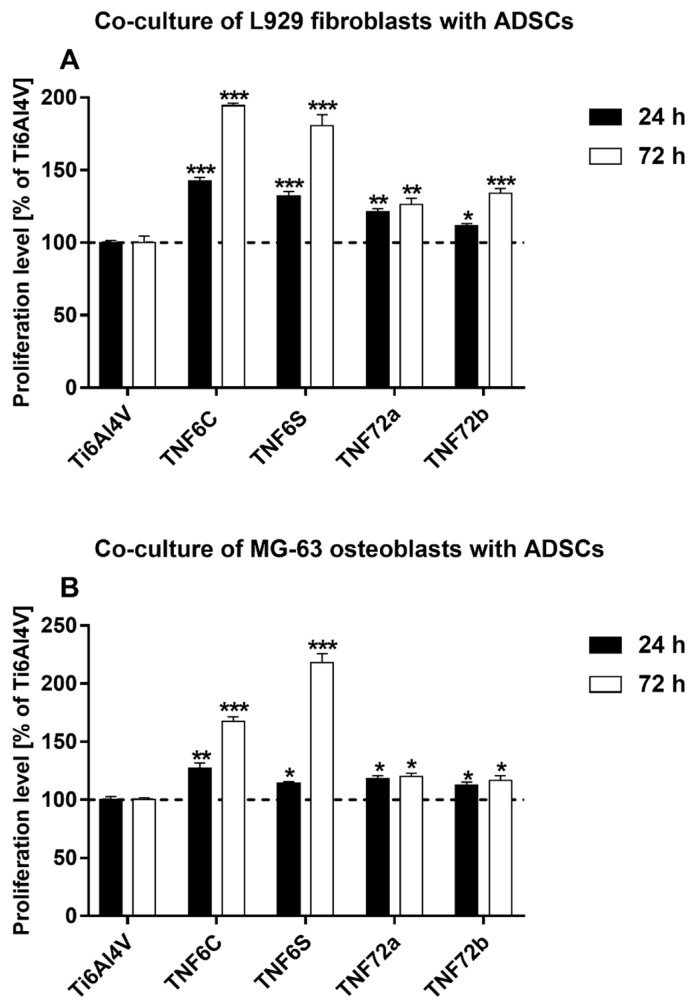
Proliferation level of L929 murine fibroblast cells (**A**) or human osteoblast-like MG 63 cells (**B**) co-cultured with human adipose-derived mesenchymal stem cells (ADSCs) on the surface of fibrous nanocoatings. Cell viability level was assessed using the MTT assay after 24 and 72 h of culture. The results are expressed as percentage of the both co-cultured cells cultivated on the reference Ti6Al4V alloy foils (served as 100%). The percentage values are expressed as means ± SEM of four independent experiments. Asterisks indicate significant differences at the appropriate time (after 24 or 72 h) between the cells co-cultured on the surface of the tested specimens in comparison with the reference Ti6Al4V alloy foils (Ti6Al4V) (*** *p* < 0.001, ** *p* < 0.01; * *p* < 0.05). The horizontal lines show the proliferation level of the both co-cultured cells growing on the reference Ti6Al4V alloy foils.

In the next study, we also assessed the viability of L929 fibroblasts or MG-63 osteoblasts co-cultured with ADSCs growing on the surface of the tested specimens. For this experiment, we selected the nanocoatings, which have shown the highest cell survival rate for all three tested cell lines. The results are expressed as percentage of the both co-cultured cells cultivated on the reference Ti6Al4V alloy foils (served as 100%). As can be seen in Figure 6, all tested specimens induced a significant increase in L929 fibroblasts (Figure 6A) as well as MG-63 osteoblasts (Figure 6B) viability co-cultured with ADSCs. This phenomenon was observed both after 24 and 72 h of incubation time. The highest cell viability was observed for TNF6C and TNF6S nanolayers after 72 h. The viability of L929 fibroblasts co-cultured with ADSCs then reached 194.4 ± 1.6% and 180.6 ± 7.7%, whereas the proliferation level of MG-63 osteoblast co-cultivated with ADSCs was 167.4 ± 4.1% and 218.2 ± 7.6%, respectively (*p* < 0.001). These results proved that ADSC-mediated functionalization of nanolayers increases their appropriateness for the cell growth and suitability as scaffolds mainly for fibroblasts and osteoblasts culture.

### 2.6. Cell Morphology Analyzed by Scanning Electron Microscopy

**Figure 7:**
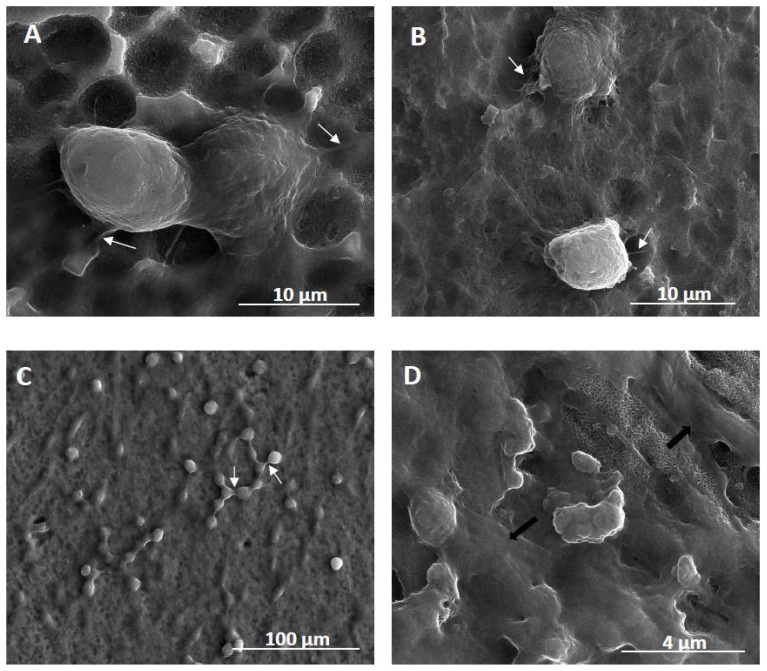
Scanning electron microscopy (SEM) images presenting the cells growing on the surface of fibrous nanocoatings. (**A**)—adipose-derived stem cells (ADSCs) “invading” the surface of TNF72a nanocoatings after 24h; (**B**)—ADSCs co-cultured with L929 fibroblasts on the TNF72a samples for 24 h; (**C**)—successful co-culture of ADSCs and L929 fibroblasts after 72h; (**D**)—successful co-culture of ADSCs and MG-63 osteoblasts after 72h. White arrows in the micrographs indicate filopodia that attach the cells to the surface (Figure 7A,B) or which are spreading between the cells (Figure 7C). Black arrows in Figure 7D show the extracellular matrix produced by the MG-63 osteoblasts.

The biointegration capacity of the selected fibrous nanocoatings in reference to growing cells morphology was evaluated by analysis of SEM micrographs (Figure 7). As it can be seen, the cells are adherent and exhibit normal morphology with cytoplasmic filopodia. These actin based cell protrusions attach the cells to the surface of nanocoatings (Figure 7A,B), while connections are also generated between the cells (Figure 7C). Moreover, ADSCs co-cultured with MG-63 osteoblasts produce huge amounts of the extracellular matrix (Figure 7D). 

## 3. Discussion

Surface properties of biomaterial are responsible for the first reactions of the body, resulting in its acceptance or rejection [57]. Numerous studies proved the influence of the topography on the osseointegration process [58,59,60]. As the roughness increases, the possibility of attaching cells, such as osteoblasts or fibroblasts, also increases, and thus the biointegration activity of the biomaterial increases [57]. Presented research confirms the positive effect of surface modification on the Sa parameter value. However, during the designing of new materials for implants, especially for long-term implants, the emphasis should be maintained on the mechanical properties of biomaterial (such us Young’s Modulus). For this purpose, the nanoindentation technique is the most suitable for testing biomaterial surfaces [61,62,63]. The mechanical properties of an implant, mainly Young’s Modulus, should be similar to that of the human bone (10–30 GPa) [64,65]. As the difference between implant and tissue properties increases, the risk of the “shielding” effect increases [65]. It has been confirmed that the occurrence of a shielding effect can cause tissue damage around the implant, bone loss, implant loosening, and premature failure of the implant [64,65,66]. According to our nanoindentation tests, the most biomechanical compatibility of tested modifications was noticed for TNF6S and TNF10C with the Young’s Modulus value beng the most similar to the value of Young’s Modulus of human cortical bone. On the other hand, the surface of the TNF6S sample has the lowest wear resistance, which negatively affects the potential biomaterial application.

The adhesion of the modified surface to the titanium alloy was determined by another novel method indicated to measure adhesion of thin coatings and used before for biomaterials applications: the nanoscratch-test [67,68]. The biomaterials surface-modified, especially for long-terms implants should be characterized by proper adhesion to the metallic substrate. The increase in coating adhesion for TNF4C-10C samples can be attributed to the decrease in the roughness of these coatings with an increase of the oxidation process time. A lower surface roughness value reduces the risk of delamination initiation on the surface, which was reported for TiO_2_ layers on titanium alloy by Gonzales et al. [69]. An increase of nanomechanical properties, such as the nanohardness (H) and Young’s modulus (E), can indicate better fracture toughness. The similar effect were reported by Kumar et al. [70]. In consequence, the tested surface with highest values of H and E (TNF72b, 7.68 GPa and 180.18 GPa) was characterized by highest adhesion to the titanium alloy (205.15 mN of critical force).

While designing a long-term implant, it is necessary to remember that the success of its use depends on the integrity of osseointegration and contact with surrounding soft tissue [71]. Therefore, it is important to give the coating, both an appropriate surface topography and mechanical properties, as well as an adequate ability to create a permanent implant-bone connection. For this purpose, in our works, TiO_2_ nanofibers, suitable for the cell growth and produced by chemical oxidation of Ti6Al4V alloy, were evaluated using three cell lines: mouse L929 fibroblasts, human osteoblasts-like MG-63 cells, and adipose-derived human mesenchymal stem cells (ADSCs). MG-63 cell line is a standard model for a bone research, which is used to study the effect of surface nanotopography on osteoblast-like cells [72]. L929 cell line belongs to the continuous cell lines of soft tissue and it is widely used to test the cytotoxicity of dental materials when employing in vitro methods of experimentation [73]. Fibroblasts are also the most common cells in the peri-implant soft tissue, which is key to the formation of the peri-implant mucosal seal and helping to prevent epithelial ingrowth [71]. ADSCs are particularly important for regenerative medicine due to their capability of differentiating into osteoblasts, and they have been reported to accelerate bone tissue healing in combination with implants [74]. Recent studies also point to the paracrine and trophic effects of mesenchymal stem cells, which allow them to influence the neighboring microenvironment and provide a new prospective on cell-based therapy and tissue regeneration [75]. The studies with the parallel use of osteoblasts, fibroblasts, and adipose-derived stem cells seemed to be a preliminary, but also a comprehensive examination of fibrous nanocoatings biointegration in vitro.

In our study, the biointegration of the tested scaffolds was examined using MTT assay (for evaluation of cell viability) and scanning electron microscopy images analysis (for assessment of cell morphology). The level of cells proliferation was assessed for the single cell line culture as well as for L929 fibroblasts or MG-63 osteoblasts co-cultured with ADSCs. Short term culture (after 24 h) of all three studied cell lines showed that titania nanofibrous layers on Ti6Al4V alloy surface were non-toxic in general and suitable for maintaining the cell growth in vitro (Figure 5A-C). However, different surface structure (anatase, rutile and amorphous ones) and wettability of samples resulted in slight differences in promoting growth of three tested cell lines. TNF72a and TNF72b specimens induced the highest viability of murine fibroblasts L929 after 24 h, whereas human MG-63 osteoblasts and ADSCs preferred the surface of TNF4C, 6C, and 10C. These differences can be explained by the surface chemical nature. The surface chemical nature preferred by fibroblasts coatings TNF72a and TNF72b resembles a spongy structure with protruding nanofibers that initiates adhesion and promotes modification with extracellular matrix proteins. The completely amorphous character of TNF72a and TNF72b favors higher viability of fibroblasts after 24 h. However, after 72 h of culture on TNF72a and TNF72b surfaces, the proliferation level of all cell lines increased as compared with the control alloy.

As we mentioned above, tested nanocoatings suitable for the cell growth were also evaluated using a cell co-culture system. This experimental approach is rarely described in the literature. However, some studies indicate that bone marrow-derived mesenchymal stem cells co-cultured with human umbilical vein endothelial cells showed enhanced osteogenesis [76]. Similarly, Birmingham and colleagues demonstrated that proliferation and osteogenic differentiation of murine bone marrow-derived mesenchymal stem cells is significantly improved when co-cultured with osteoblasts and osteocytes [77]. Recent research on articular chondrocytes co-cultured with mesenchymal stem cells gave also promising results [78]. In reference with that, we have decided to investigate the effect of ADSCs co-culture with two cell lines: L929 fibroblasts and MG-63 osteoblasts that represent cells involved in the long-term success of the implants. The co-culture experiments showed slightly different results from those described for the single cell lines. TNF6C and 6S scaffolds turned out to be the most beneficial options for the cell survival rate after 72 h (Figure 6). These coatings show crystalline properties due to the presence of rutile/anatase nanocrystals that were often reported as being toxic for cells [79]. However, ADSCs functionalize the initially unfavorable surface, prepare it for more specialized and therefore demanding cells, and make it suitable for the cell growth. Eventually, it appears to be a beneficial surface for maintaining osteoblasts and fibroblasts growth and proliferation. As seen from SEM images, the cells are adherent, exhibit normal morphology with cytoplasmic filopodia, spread on the scaffolds, and produce huge amounts of extracellular matrix (Figure 7). Filopodia as the actin based cell protrusions are one of the most important cellular sensors, which collect information on whether the surface is suitable for cell attachment and proliferation, cell-cell interaction, and also allow cell migration toward the destination [80]. The extracellular matrix (ECM) is a complex assembly of molecules that interact with one another, creating the physical microenvironment necessary for the cell to survive and to function, for cell anchorage, and for providing a tissue scaffold for cell migration [81]. Therefore, filopodia formation and ECM production is evidence of the surface biointegration activity of the tested scaffolds. Further experiments elucidating the osteogenic potential of ADSCs and cytophysiology of osteoblasts on the fibrous nanocoatings are ongoing.

## 4. Materials and Methods

### 4.1. Synthesis of Titania Nanocoatings

#### 4.1.1. Titania Nanofibers (TNF4-10)

The chemical oxidation method of titanium alloy ((Ti6Al4V, grade 5 foil, 99.7% purity, 0.20 mm thick, Strem Chemicals, Inc. (Bischheim, France), 6 mm × 60 mm pieces)) was used to produce the titania nanofiber coatings (TNF4-10). Ti6Al4V samples were polished and then were sonicated in acetone, ethanol and water (15 min in every liquid). The surface of the substrates were chemically etched in a 1:1 mixture of concentrated HCl and H_2_O at 353 K for 30 min, cleaned with deionized water, and dried in Argon stream. After acid treatment, titanium surfaces was heated in 30% H_2_O_2_ solution at 358 K, for different oxidation times, i.e., *t* = 4, 6, and 10 h. Samples were heated in two ways: (a) in an incubator (TNF4S-10S), (b) under a reflux condenser (TNF4C-10C). After chemical oxidation was completed, the samples were ultrasonically cleaned in deionized water and acetone, and then were dried in Argon stream. 

#### 4.1.2. Titania Nanofibers (TNF72)

In order to fabricate titania nanofibers layers (TNF72) on the surface of Ti6Al4V substrates, the chemical oxidation method was applied ((Ti6Al4V, grade 5 foil, 99.7% purity, 0.20 mm thick, Strem Chemicals, Inc. (Bischheim, France), 6mm × 60 mm pieces)). Substrate samples were polished and then were sonicated in acetone, ethanol and water. Samples were etched in two ways at ambient temperature: (a) in 2 M HF solution for 10s (TNF72a), (b) in a 1:4:5 mixture of HF:HNO_3_:H_2_O for 30 s (TNF72b). After this, the samples were cleaned with deionized water and dried in Argon stream. Next, Ti6Al4V samples were immersed in 30 wt% H_2_O_2_ solution. The reactants were kept at 358 K in an oven for 72 h. After chemical oxidation was completed, the samples were ultrasonically cleaned in deionized water and acetone, and then they were dried in Argon stream. 

### 4.2. Characterization of Titania Nanocoatings

#### 4.2.1. Structure and Morphology Characterization 

Surface morphology of all samples was studied using a Quanta scanning electron microscope with field emission (SEM, Quanta 3D FEG, Huston, TX, USA). The structure of the produced coatings was estimated using X-ray diffraction (PANalytical X’Pert Pro MPD X-ray diffractometer using Cu-Kalfa radiation, grazing incidence angle mode–GIXRD; the incidence angle was equal to 1 deg). Surface topographies were studies using Atomic Force Microscopy (AFM, NaniteAFM, Nanosurf UK, Bracknell, UK) with non-contact mode with 55 nN force and an area of 50 × 50 µm.

#### 4.2.2. The Wettability and Surface Free Energy 

The application of the goniometer (DSA 10 Krüss GmbH, Hamburg, Germany) with drop shape analysis software (ADVANCE), allowed measuring the contact angle with the use of the droplet method. Based on obtained results, the wettability of the coatings was estimated. In order to determine the free surface energy, a mathematical calculation using the Owens-Wendt method was applied. For this purpose, it was necessary to measure the contact angle using two liquids: polar distilled water and non-polar diiodomethane. The volume of the distilled water drop in the contact angle measurement was 3 µL and in the case of diiodomethane 4 µL. Each measurement was carried out three times immediately after deposition of the drop.

#### 4.2.3. Mechanical Properties

The nanomechanical properties such as nanoindentation and adhesion determined by nanoscratch-test were performed with the nanoindenter NanoTest™ Vantage (Micro Materials Ltd., Wrexham, UK) using a Berkovich three-sided pyramidal diamond with angle 124.4°. Twenty-five independent measurements of nanoindentation were performed for each tested sample. The maximum force was 50 mN with the loading time 15 s, unloading 10 s and 5 s dwell with maximum force. The distance between each measurement was 20 µm. The surface hardness (H) and Reduced Young’s modulus (Er) were determined by the Oliver and Pharr method. To calculate Young’s modulus (E) from Reduced Young’s modulus, we used a Poisson’s ratio of 0.3. The adhesion of the layers was determined by nanoscratch-test with applied force from 0 to 200 mN at a loading rate of 1.3 mN/s on a distance of 500 µm. For all tested specimens, we performed 10 independent measurements. The adhesion of the layers was assessed based on the observation of an abrupt change in frictional force during the test. 

### 4.3. Biological Studies

#### 4.3.1. Cell Culture

Human osteoblast-like MG 63 cells (European Collection of Cell Cultures, Salisbury, UK, cat. no. 86051601) were cultured at 37 °C in 5% CO_2_ and 95% humidity in Eagle’s Minimum Essential medium (EMEM) containing 2 mM l-glutamine, 1 mM sodium pyruvate, MEM non-essential amino acid, heat-inactivated 10% fetal bovine serum (FBS), 100 µg/mL streptomycin, and 100 IU/mL penicillin (all compounds from Sigma-Aldrich, Darmstadt, Germany). The cells were passaged using 0.25% trypsin-EDTA solution (Sigma-Aldrich) when reaching 70–80% of confluency.

L929 murine fibroblast cells (American Type Culture Collection) were cultured at 37 °C in a humidified atmosphere with 5% CO_2_. Culture medium consisted of RPMI 1640 medium containing 2 mM l-glutamine (Sigma-Aldrich, Darmstadt, Germany), 10% heat-inactivated fetal bovine serum (FBS), 100 IU/mL penicillin and 100 µg/mL streptomycin (PAA Laboratories GmbH, Cölbe, Germany). L929 cells were passaged when reaching 70–80% confluency using cell scraper. 

Adipose-derived human mesenchymal stem cells (ADSC) were purchased from PromoCell and cultured at 37 °C in humidified atmosphere containing 5% CO_2_. According to the manufacturer’s protocol, the culture medium consisted of Mesenchymal Stem Cell Growth Medium^®^ and 10% Supplement Mix^®^ (PromoCell GmbH, Heidelberg, Germany), with 100 U/mL of penicillin and 100 µg/mL streptomycin (Sigma-Aldrich, Darmstadt, Germany). The cells were passaged using 0.04% trypsin-EDTA solution (Sigma-Aldrich) when reaching 70–80% of confluency.

#### 4.3.2. Cell Proliferation Assays

The effect of the TiO_2_ nanofibers, produced by chemical oxidation of Ti6Al4V alloy on the cell proliferation (measured after 24 and 72 h) was studied using the MTT (3-(4,5-dimethylthiazole-2-yl)-2,5-diphenyl tetrazolium bromide; Sigma Aldrich, Darmstadt, Germany) assays. Firstly, we have studied the proliferation level of all tested cell lines growing on the surface of substrates. MG-63 osteoblasts, L929 fibroblasts and ADSCs were seeded at a density of 1 × 10^4^, 1 × 10^4^, and 3 × 10^4^ cells/well, respectively, onto the autoclaved nanolayers placed in 24-well culture plates and then cultured for 24 and 72 h. 

In the separate experimental set, we investigated the effect of fibrous nanocoatings on the proliferation level of MG-63 osteoblasts or L929 fibroblasts co-cultured with adipose-derived mesenchymal stem cells. ADSCs were seeded on the sterile nanolayers (3 × 10^4^ cells in a 10-µL drop) and left for 4 h to adhere. Then, MG-63 osteoblasts or L929 fibroblasts at a density of 1 × 10^4^ cells/well were seeded on the tested specimens covered with mesenchymal stem cells and incubated for 24 and 72 h. The cells in the co-culture system were cultivated in a suitable complete culture medium: EMEM for MG-63 osteoblasts and RPMI 1640 for L929 osteoblasts. 

After the respective incubation time, the substrates were rinsed with phosphate buffered saline (PBS) and transferred to a new 24-well culture plate. MTT solution (5 mg/mL; Sigma-Aldrich) in a suitable culture medium without phenol red was added to each well and the plates were incubated for 3 h. Then, the MTT solution was aspirated and 500 μL of dimethyl sulfoxide (DMSO; 100% *v*/*v*; Sigma Aldrich) was added to each well. Finally, the plates were shaken for 10 min. The absorbance was measured at the wavelength of 570 nm with the subtraction of the 630 nm background, using a microplate reader (Synergy HT; BioTek, Winooski, VT, USA). The blank groups (the plates incubated without the cells) were treated with the same procedures as the experimental groups. All measurements were done in duplicate in four independent experiments. The results were expressed as percentage of control cells, which served as 100% at the respective incubation time. For the assessment of the proliferation level of the single cell lines, MG-63 osteoblasts, L929 fibroblasts, or adipose-derived mesenchymal stem cells growing on the Ti6Al4V reference alloys were used as control cells. In the case of a co-culture system, both co-cultured cells cultivated on the Ti6Al4V reference alloys served as control samples (100%). 

#### 4.3.3. Cell Morphology Observed by Scanning Electron Microscopy

The analysis of morphological changes of MG-63 osteoblasts or L929 fibroblasts co-cultured with adipose-derived mesenchymal stem cells growing on the surface of the selected fibrous nanocoatings was performed using the scanning electron microscopy (SEM; Quanta 3D FEG; Carl Zeiss, Göttingen, Germany). The cells were incubated on the tested specimens for 24 or 72 h. Then, the specimens were rinsed with PBS to remove the non-adherent cells and fixed in 2.5% *v/v* glutaraldehyde (Sigma-Aldrich) for a minimum of 4 h (maximum 1 week). After that, the plates were washed again with PBS and dehydrated in a graded series of ethanol concentration (50%, 75%, 90%, and 100%) for 10 min. Finally, the specimens were dried in vacuum-assisted desiccators overnight and stored at room temperature until the SEM analysis was performed. 

#### 4.3.4. Statistical Analysis in the MTT Assay

All values are reported as means ± standard error of the means (SEM) and were analyzed using the nonparametric Kruskal–Wallis one-way ANOVA test with the level of significance set at *p* < 0.05. Statistical analyses were performed with GraphPad Prism 7.0 (La Jolla, CA, USA). 

## 5. Conclusions

The direct oxidation method of Ti6Al4V substrates led us to produce the hydrophobic fibrous TiO_2_ scaffolds (TNF) on their surface. The amorphousness of TNF72 samples and the polycrystalline structure of TNF4-10 ones have been proven by X-ray diffraction studies. Mechanical properties and the biointegration activity of surface-modified samples turned out to be closely linked with chemical structure of coatings, their wettability, and nanotopography. In comparison to TNF4-10 samples, the TNF72 scaffolds characterize better adhesion to the titanium alloy surfaces, higher values of the Young’s Modulus, and lower roughness. The viability level of all cell lines (mouse L929 fibroblasts, human osteoblasts-like MG-63 cells, and adipose-derived human mesenchymal stem cells (ADSCs)) increased after 72 h of culture on completely amorphous TiO_2_ nanofibers surfaces (TNF72) versus the control sample (Ti6Al4V alloy). The co-culture experiments showed slightly different results from that described for the single cell lines. ADSCs have demonstrated the ability to functionalize the initially unfavorable surface, making it appropriate for the cell growth and preparing it for more specialized and demanding cells. The presented data allow us to expect that titania nanofiber scaffolds will prove to be beneficial and can be applied as a novel alternative for bone tissue regeneration.

## Figures and Tables

**Table 1:** Nanomechanical and nanoindentation properties of the reference Ti6Al4V and of titanium nanocoatings.

Biomaterial Sample	Hardness H (GPa)	Young’s Modulus E (GPa)	Maximum Depth of Indentation (nm)	H/E (−)	H^3^/E^2^ (GPa)
Ti6Al4V	10.94 ± 1.42	212.48 ± 16.69	472.25 ± 26.11	0.0513 ± 0.0034	0.0294 ± 0.0078
TNF4S	6.68 ± 2.59	170.85 ± 58.26	604.76 ± 85.71	0.0388 ± 0.0039	0.0105 ± 0.0057
TNF6S	3.92 ± 1.73	133.85 ± 42.12	800.94 ± 191.73	0.0285 ± 0.0068	0.0039 ± 0.0033
TNF10S	4.59 ± 1.41	136.54 ± 27.81	718.59 ± 112.31	0.0330 ± 0.0047	0.0054 ± 0.0028
TNF4C	5.43 ± 2.15	166.34 ± 51.06	669.96 ± 127.95	0.0322 ± 0.0056	0.0063 ± 0.0044
TNF6C	6.00 ± 2.00	165.11 ± 39.18	634.01 ± 101.29	0.0356 ± 0.0046	0.0083 ± 0.0048
TNF10C	4.69 ± 1.50	133.22 ± 20.80	709.13 ± 88.34	0.0348 ± 0.0054	0.0064 ± 0.0050
TNF72a	6.27 ± 0.88	170.92 ± 15.73	601.98 ± 41.46	0.0356 ± 0.0021	0.0085 ± 0.0021
TNF72b	7.68 ± 1.78	180.18 ± 27.40	561.09 ± 80.11	0.0421 ± 0.0044	0.0143 ± 0.0053

**Table 2:** Adhesion properties of the titanium nanocoatings to the titanium alloy surfaces.

Biomaterial Sample	Nanoscratch-Test Properties	
	Critical Force (mN)	Critical Friction Force (mN)
TNF4S	164.20 ± 61.12	131.68 ± 52.66
TNF6S	107.40 ± 27.27	91.86 ± 22.80
TNF10S	116.69 ± 28.67	90.69 ± 21.53
TNF4C	130.95 ± 47.15	104.83 ± 47.34
TNF6C	139.03 ± 34.59	105.91 ± 32.16
TNF10C	140.91 ± 34.10	117.33 ± 42.05
TNF72a	203.91 ± 37.59	183.24 ± 61.29
TNF72b	205.15 ± 48.96	140.27 ± 46.27

## Supplementary Materials

The following are available online at https://www.mdpi.com/1422-0067/20/22/5642/s1, Table S1: Results of contact angle and surface free energy (SFE) measurements; contact angles were measured three times using distilled water and diiodomethane; SFE values were determined using the Owens-Wendt method.
