# Supplementary material for: Titania Nanofiber Scaffolds with Enhanced Biointegration Activity—Preliminary In Vitro Studies
Source: Int J Mol Sci. 2019 Nov 11;20(22):5642. doi: 10.3390/ijms20225642 (PMC6888681; doi:10.3390/ijms20225642)
Supplement: Supplementary file 1 [file ijms-20-05642-s001.pdf]

Table S1. The results of contact angle and surface free energy (SFE) measurements; contact angles were measured three times using distilled water and diiodomethane; the SFE values were determined using the Owens-Wendt method.

	Average contact angle [°] ± standard deviation		Surface free energy ± standard deviation [mJ/m²]
	Measuring liquid		
**Biomaterial sample**	**Water**	**Diiodomethane**	
Ti6Al4V	81.3 ± 0.2	49.2 ± 0.9	38.5 ± 0.3
TNF4S	99.5 ± 0.0	61.0 ± 0.9	27.6 ± 0.2
TNF6S	104.9 ± 0.1	61.9 ± 1.1	27.6 ± 0.3
TNF10S	106.8 ± 0.3	48.0 ± 1.2	37.4 ± 0.3
TNF4C	97.5 ± 0.1	52.0 ± 0.7	32.9 ± 0.2
TNF6C	109.5 ± 0.2	48.0 ± 1.7	38.4 ± 0.5
TNF10C	113,8 ± 0.3	39.4 ± 1.0	46.4 ± 0.3
TNF72a	98.2 ± 0.0	59.5 ± 1.3	28.4 ± 0.3
TNF72b	108.5 ± 0.3	46.2 ± 0.6	39.3 ± 0.2
